# Pharmaceutical Chemistry

**Published:** 1845-01

**Authors:** 


I. THE PHARMACOPŒIA OF THE ROYAL COLLEGE OF PHYSICIANS OF EDINBURGH. 12mo. pp. 217. Edinburgh, 1839.

The Second Edition of the above, 1841.

II. A DISPENSATORY, OR COMMENTARY ON THE PHARMACOPŒIAS OF GREAT BRITAIN, &c. &c. By *Robert Christison*, M.D. F.R.S.E. Professor of Materia Medica in the University of Edinburgh, Vice-President of the College of Physicians, &c. 8vo. pp. 978. Edinburgh, 1842.

III. THE LONDON DISPENSATORY, &c. &c. By *Anthony Todd Thomson*, M.D. F.L.S. Professor of Materia Medica, Therapeutics and Medical Jurisprudence in University College, London, Fellow of the Royal College of Physicians in London, &c. &c. 8vo. pp. 1317. London, 1844.

THE extraordinary progress which has been made in chemical science in general, and more especially in organic chemistry, within a few years, has greatly increased the necessity for more frequent revisions of those national depositories of medicinal preparations—the Pharmacopœias of Great Britain.

This examination was more especially called for with respect to the Edinburgh Pharmacopœia, an edition of which had not appeared for about twenty-two years, until the present was published; the propriety of this step on the part of the College must be universally admitted, if it were for the purpose alone, of introducing those important medicines the vegetable alkalis, with directions for their preparation and that of their saline combinations.

We now propose to examine somewhat minutely the Edinburgh Pharmacopœia, which made its appearance under the auspices of the then President of the College, Dr. Christison—a name known throughout Europe, and as widely respected, as the author of an excellent *Treatise on Poisons.*

Since the appearance of the Pharmacopœia Dr. Christison's and Dr. Thomson's Dispensatories have also both been published, and to these works we shall have occasion to make frequent reference.

We shall offer no apology either to Dr. Christison or Dr. Thomson for the free observations which may arise during the proposed examination of their works; and from the time of Nich. Culpeper to the present, pharmacopœias, like other public acts, have been considered fair objects for criticism, and no one will deny the truth of the trite observation, that the errors which are committed or sanctioned by authority are doubly dangerous.

Dr. Christison has shown, by his numerous remarks on the London Pharmacopœia, that works of this description are not to be excepted from the lash of criticism, and Dr. A. T. Thomson, in referring to the same work, says, in a former edition of his Dispensatory, that the London Pharmacopœia is "far from being worthy of the present advanced state of medical science;" a censure so indiscriminate and sweeping as this, naturally challenges inquiry into the nature of its author's criticisms, in order to ascertain their just value.

There are a few observations in the Preface of the Edinburgh Pharmacopœia requiring notice; the College say, "in the plan of the present Pharmacopœia we have thought it advisable to deviate materially in several respects from those of former years."

"That we have departed from all previous practice of Colleges in this country by publishing our Pharmacopœia in the English language is an alteration, which, as it has been sanctioned by the almost unanimous consent of the College, will also, we apprehend, meet with the general approbation of the medical public. The time is perhaps gone by when public opinion required as a test of learning that a College of Medicine should write in Latin alone; and it may even be questioned whether the practice be not open to censure as leading to risks of inaccuracy in preparing and compounding drugs. Besides, the favourable reception of unauthorized translations of former Pharmacopœias, together with the slow sale of the last Latin Edition of 1817, seemed sufficiently to indicate the wishes of the profession on this subject, and to show that the Latin language cannot be any longer retained, without occasioning, as of late, serious delays and obstructions in the way of future improvement."—*Preface*, p. viii.

Without questioning the propriety of the above-described change, we may remark, that it has been but partially carried into effect; for not only are the Latin names of the articles of the *Materia Medica* retained, but even those of the *Preparations and Compounds*, and the latter, indeed, without being translated;—thus, to take the first preparation under the head of *Acids*, we have *Acetum Destillatum* without the English, and this incongruity pervades the whole work; it is indeed true that there is an English Index, in which there occurs *Vinegar, distilled*, p. 47; but it must surely be admitted, that in a Pharmacopœia professedly English, the Index is not the only proper place to find it.

The College afterwards remark that "the increasing frequency and extent of the adulteration of drugs induced us to propose, a few years ago, to the Royal College of Physicians of London, in the course of certain negociations relative to an Imperial or General Pharmacopœia for the Empire, that to the List of the Materia Medica there should be added a short statement of characters for ascertaining, that the leading articles are free from known sophistications, and of the due degree of purity for medical use. The suggestion has been partly adopted in the recent edition of the London Pharmacopœia; and in the present work we have endeavoured to carry more completely into effect the principle we propounded."

It will be naturally concluded, by those who read this statement, that the very useful proposal which it contains actually originated with the Edinburgh College; it is extremely probable that both Colleges borrowed the idea from the Prussian Pharmacopœia of 1829.

In our present undertaking we shall not commence with the first preparation that the Pharmacopœia contains; this is the less requisite, because certain portions of the work have already been examined in two contemporary publications,* and, having stated thus much, we now proceed to offer some observations on various preparations and on the remarks which have been made upon them in the Dispensatories of Drs. Christison and Thomson.

*Acidum Tartaricum.*—It having been shown that the Edinburgh College in the first edition of their English Pharmacopœia had committed a great error in the formula for preparing this acid by forgetting that the dilute sulphuric acid of the London College is of a different strength from their own, this mistake is corrected in the 2nd edition. There are, however, some remarks of Dr. Christison's on this acid, and on the London Pharmacopœia, which demand notice; he says (p. 51) of tartaric acid, that "soluble earthy salts form in its solution white precipitates, which are earthy tartrates." Doubting the accuracy of this statement, we mixed solutions of tartaric acid with those of the following salts: chloride of calcium, sulphate of lime, sulphate of magnesia, nitrate of barytes, nitrate of strontia and alum, but no precipitation whatever took place in any one of them. It is indeed true that lime and barytes form insoluble compounds with tartaric acid, when the simple solutions of these earths are saturated with a solution of the acid, but when their *salts* are added to the acid, either no tartrate is formed, or it is held in solution by the acid which the tartaric has set free.

---

* Medical Gazette and Pharmaceutical Journal.

We shall now notice, once for all, one of a series of criticisms on the London Pharmacopœia which here appears for the first time, but which is with occasional variations of language repeated about thirty times in Dr. Christison's work: speaking of the London College, and of the tests which they have employed for ascertaining the purity of tartaric acid.—Dr. C. says (p. 51) " the effect of the salts of potash in separating from its solution a bitartrate of potash,—which the same College has annexed,—is a test of its nature, and not of its purity." Now unless the nature of a substance be known, how are we to judge of its purity? On referring to Benzoic Acid in the Edinburgh Pharmacopœia we find "Tests.—*Edin.* Colourless: sublimed entirely by heat," and with respect to arsenious acid, we meet with "Tests.—*Edin.* Entirely sublimed by heat;" now as arsenious acid is usually as colourless as benzoic acid, it is evident that, according to the Edinburgh plan, they may be regarded as identical, unless some proofs of their nature be also admitted, for both will answer to the tests proposed.

With respect to Tartaric Acid, two errors, but not very important ones, occur in Dr. Thomson's Dispensatory. In p. 860, it is stated that, " the use of the hydrochloric acid is to form with the chalk a chloride of *lime*," which ought to be of *calcium*, and it is mentioned that " 100 grains of the crystallized acid, in solution, saturate 200 grains of crystallized carbonate of soda," now as the equivalents of these substances are stated to be 75·48 and 143·42 respectively, the carbonate of soda required is 190 grains, for 75·48 : 143·42 : : 100 : 190.

*Ammoniæ Aqua*, et *Ammoniæ Aqua fortior.*—Before we proceed to consider these important preparations, we shall offer a few observations on the extreme caprice both of the College and Dr. Christison, with respect to some points of nomenclature regarding hydrochlorate of ammonia. In the Materia Medica of the first edition of the Pharmacopœia we find this salt thus described :—Ammoniæ murias. *Hydrochlorate of ammonia*, and there are three preparations in which it is employed, viz. Ammoniæ Aqua, Ammoniæ Carbonas, and Spiritus Ammoniæ; in the directions for obtaining the first of these, it is termed Muriate of ammonia, in the second Sal-ammoniac, a name which it does not bear in the Materia Medica, while in the third, the College recur to Muriate of ammonia. In the second edition of the Pharmacopœia we find Ammoniæ murias, Hydrochlorate of ammonia, *Sal-ammoniac;* in this edition the last term is used in the directions for the second preparation above-named, while, in the first and third, muriate of ammonia is employed.

Dr. Christison has not much tended to dissipate the inconsistencies which I have pointed out, for where the College, under Ammoniæ Aqua, say Muriate of Ammonia, he has Sal-ammoniac; for under Ammoniæ Carbonas both also use Sal-ammoniac instead of Muriate of Ammonia.

The following are the directions of the Edinburgh Pharmacopœia for preparing by one operation both solutions of ammonia, one of specific gravity 880, and the other of 960.

" Take of Muriate of ammonia, thirteen ounces;
Quicklime, thirteen ounces;
Water, seven fluidounces and a half;
Distilled water, twelve fluidounces.

Slake the Lime with the water, cover it up till it cool, triturate it well and quickly with the Muriate of ammonia previously in fine powder, and put the mixture into a glass retort, to which is attached a receiver with a safety-tube. Connect with the receiver a bottle also provided with a safety-tube, and containing four ounces of the Distilled water, but capable of holding twice as much. Connect this bottle with another loosely corked, and containing the remaining eight ounces of distilled water. The communicating tubes must descend to the bottom of the bottles at the further end from the retort; and the receiver and bottles must be kept cool by snow, ice, or a running stream of very cold water. Apply to the retort a gradually increasing heat till gas ceases to be evolved; remove the retort, cork up the aperture in the receiver where it was connected with the retort, and apply to the receiver a gentle and gradually-increasing heat, to drive over as much of the gas in the liquid contained in it, but as little of the water as possible. Should the liquid in the last bottle not have the density of 960, reduce it with some of the Stronger Aqua ammoniæ in the first bottle, or raise it with distilled water, so as to form Aqua ammoniæ of the prescribed density."

We candidly and unhesitatingly admit that we are unable to comprehend these directions, and consequently have not attempted to reduce them to practice; we will therefore only remark that the quantity of lime directed to be used is inconveniently and unnecessarily large.

By what appears to be a mere typographical error, Dr. Christison states that ammonia was discovered by Dr. Priestley in the pure gaseous state in 1790; the fact is that he described it in the first volume of his Experiments on different kinds of Air, in 1774.

In page 101 of his Dispensatory, Dr. Christison says, "TESTS, *Edin. Lond.* Density 880° (882 at 62°, L.); one fluidounce with two fluid ounces and a half (three L.) of water, makes the Aqua or Liquor Ammoniæ."

In page 106, with reference to this part of the subject he observes, "the weaker solution of ammonia of the density 960 may be made at once from the stronger by adding to it, as the London College directs, three times its volume of water. Rather less water, however, is required; for one volume of solution at 880 with three volumes of water has a density of 985. The Edinburgh College directs two volumes and a half of water to be used."

Dr. Christison might have known that the error of directing three volumes of water instead of two for diluting the stronger solution of ammonia had been pointed out and corrected long before his work appeared; in the above quotation he states inaccurately the density of a mixture of three volumes of water and one volume of solution of ammonia of sp. gr. 880; these mixtures are nearly of a mean density, and that just mentioned is of sp. gr. 970 and not 985; and moreover, a mixture of one volume of 880, with $2\frac{1}{2}$ volumes of water, has a density of 965 instead of 960, as directed by the Edinburgh College; two volumes only of water should be used.

We have stated why we have not prepared the solutions of ammonia according to the Edinburgh formula, we shall, however, make a few observations on the results obtained by Dr. Christison by employing it. He tells us (p. 103) that, by "using one-fourth of the materials mentioned in the College formula," he obtained in the

| | | | | | | | | |
|---|---|---|---|---|---|---|---|---|
| Second receiver | 712 grains | of Solution | of Ammonia, | sp. gr. | 880 |
| Third | do. | 975 | „ | „ | „ | „ | 960 |
| First | do. | 835 | „ | „ | „ | „ | 971 |

According to Davy's table, quoted by Dr. Christison, the Solution in the

| | | | | | | |
|---|---|---|---|---|---|---|
| Second receiver contained nearly | 31 | per cent. of Ammonia | | = | 220 grains. |
| Third „ „ | 10 | do. | do. | = | 97 „ |
| First „ „ | 7.2 | do. | do. | = | 60 „ |
| Total Ammonia obtained | | | | | 377 grains. |

One fourth of 13 ounces amounts to 1560 grains, and from this quantity of hydrochlorate of ammonia, which contains 490 grains of the alkali, only 377 were thus obtained, the loss of 113 grains, or nearly one-fourth of the whole, occurring notwithstanding the use of two safety-tubes, and of three receivers "kept cold by snow, ice, or a running stream of very cold water."

Dr. Christison further observes that, "in the trial of the Edinburgh method mentioned above, the product in the last two receivers obtained from three ounces and ten drachms avoirdupois of sal-ammoniac, when brought to the uniform density of 960, measured nine fluidounces and a-half; which account for 82 per cent. of the whole ammonia in the salt." Now 9½ fluidounces of solution of ammonia of sp. gr. 960 weigh 3,990 grains, which at 10 per cent. = 399 ammonia, but we have shown that the products of the second and third receiver amount to only 317 grains, and consequently they could form only 7·55 fluidounces of 960 instead of 9·5.

Dr. Christison calculates that the quantity of ammonia which he obtained in 9½ fluidounces of solution at 960, amounts to 82 per cent. of that contained in the hydrochlorate of ammonia employed by him; of this salt he used 3 ounces and 10 drachms avoirdupois, or 1586 grains, (containing 500 of ammonia), instead of 3¼ ounces troy, or 1560 grains; nine fluidounces and a-half of solution of ammonia of 960 contain 400 of ammonia certainly amounting to 80 per cent. of that contained in the salt; but we have shown that the whole contents of the second and third receivers contained only 317 of ammonia, amounting to less than 64, instead of 82 per cent. of the sal-ammoniac used.

In copying the Edinburgh formula Dr. Thomson has made a mistake, for the occurrence of which it is somewhat difficult to account; in the original, the water to be used is thus stated:—"Water, seven fluidounces and a-half; Distilled water, twelve fluidounces," which quantities are thus altered by Dr. T., "water, *seven fluidounces* and *a-half;* distilled water, *one pound;* water, *twelve fluidounces.*"

Dr. Thomson concludes the formula of the Edinburgh College for preparing the solutions of ammonia, with the following statements. "The specific gravity of this solution of ammonia is to that of distilled water as 0·939 to 1000. It should be preserved in small vials well stopped." Now by the formula *two* solutions of ammonia of very different densities are directed to be prepared, one of density 880, and the other of 960, so that the density of 0·939 refers to neither. In fact, the passage above quoted is part of the formula of the Pharmacopœia of 1817, and was very properly contained in former editions of the London Dispensatory, but is out of place in the present.

Referring to solution of ammonia of density 0·960, Dr. Thomson says, "at 46° Fahr. the ammonia in this solution crystallizes, and at 68° the fluid assumes the appearance of a thick jelly." These statements also appeared in the eighth and ninth editions, but in p. 188 of the latter we find it more correctly stated that the solution "forms a gelatinous mass when it is cooled down to 40° *below zero*."

*Ammoniæ Acetatis Aqua.*—"The Spirit of Mindererus," Dr. Christison says, "should always be made with distilled vinegar, and not, as many druggists are in the practice of doing, with pyroligneous acid sufficiently reduced, for when made in the latter way its taste is more disagreeable, and it is more apt to spoil."

If by pyroligneous Dr. Christison means as its name correctly imports impure acetic acid obtained from the destructive distillation of wood, we admit the accuracy of that part of the foregoing statement which relates to disagreeable taste; but, if the acetic acid be prepared as it is in London, though procured from pyroligneous acid, the preparation is more pleasant than that made with distilled vinegar, for it is quite free from empyreuma, and we have kept it for about five months, under unfavourable circumtances, without its undergoing any change.

In reference to the variable strength of this preparation Dr. Christison remarks: "It will be apparent from what has been said under the head of Acetic Acid that, as vinegars differ exceedingly in point of strength, so may distilled vinegars, unless attention be paid to correct the product according to its density. Distilled vinegar, taken as just distilled, will be found to be occasionally three times as strong as in other samples."

For argument's sake we will admit the accuracy of this statement, and if it happen that the product of a distillation should be three times as strong as the standard, then indeed it might be corrected by the addition of water; but if it should occur, and it is much more likely to do so, that the product is weaker than the standard, we do not see how any correction can be made. In fact, supposing the statement to be correct, it is impossible to adduce a better argument for the substitution of diluted acetic acid for distilled vinegar.

The following are the directions of the London Pharmacopœia for this preparation.

> Take of Sesquicarbonate of Ammonia four ounces and a half,
> or as much as may be sufficient,
> Distilled Vinegar four pints,
> Add the Sesquicarbonate of Ammonia to the Vinegar to saturation.

In reference to this formula Dr. Christison says "that, if the London College be right on the saturating power ascribed to its distilled vinegar as determined by carbonate of soda, the proportion of sesquicarbonate of ammonia recommended in the present process must be wrong. If thirteen grains of carbonate of soda neutralize a hundred grains of their distilled vinegar, then four ounces and a-half of sesquicarbonate of ammonia are more than enough to saturate four pints of it."

The saturating power of the distilled vinegar is to be determined in the present case by that of sesquicarbonate of ammonia, which, being subject to some variation, the directions are "add the sesquicarbonate of ammonia to the vinegar to saturation."

If however these directions are to be understood as Dr. Christison would interpret them, what is the meaning of the following words of the Edinburgh College in the formula for preparing Citric Acid? "Take of lemon-juice four pints; prepared chalk four ounces and a-half, or a sufficiency." Do the Edinburgh College put the same construction on these words that Dr. Christison does upon the similar expressions of the London College? Certainly not, for they say, "add the chalk to it [the lemon-juice] while hot by degrees till there is no more effervescence, and the liquor ceases to taste acid," in other words, "to saturation." In point of fact,—both Colleges employ a definite quantity of the acids, and an indefinite portion of the bases in the first instance, saturation determining the exact quantity.

But let us now examine the mode in which the Edinburgh College directs the preparation of Ammoniæ Acetatis Aqua:—

"Take of Distilled Vinegar (from French Vinegar in preference) twenty-four fluidounces;
Carbonate of Ammonia, one ounce.

Mix them and dissolve the salt. If the solution has any bitterness, add by degrees a little distilled vinegar till that taste be removed."

Following the plan adopted by Dr. Christison of determining the saturating power of vinegar with respect to sesquicarbonate of ammonia by that which it possesses as to carbonate soda, we find it stated, in the Materia Medica, that 100 minims of distilled vinegar of sp. gr. 1005 neutralize 8 grains of carbonate of soda, consequently, 24 fluidounces, or 11,520 minims, saturate 921 grains of the carbonate, equivalent to 377 only of sesquicarbonate of ammonia (144 : 59 : : 921 : 377), whereas the College order 480 grains of the ammoniacal salt to be used at once, without any attempt to determine the state of saturation; the directions are, "mix them and dissolve the salt"—not "to saturation," as in the London Pharmacopœia.

The excess of carbonate of ammonia amounts to 103 grains, or more than one-fifth of the whole quantity used; it is indeed true that "a little distilled vinegar" is to be added by degrees to remove any bitterness which the solution may possess, but it could hardly have occurred to the College that this little must amount to about one-fourth of that which had already been employed.

We will venture a little further in examining the reasonableness of Dr. Christison's criticism, by inquiring what would have been the excess of carbonate of ammonia in the London formula, supposing that it had directed, like the Edinburgh College, the whole of the vinegar and ammoniacal salt to be mixed at once, without examination as to the state of saturation. As 100 grains of the London distilled vinegar saturate 13 grains of carbonate of soda, and supposing the density to be 1006, four pints must weigh 35,210 grains, and saturate 4,577 grains of carbonate of soda, or 1875 grains of sesquicarbonate of ammonia, the excess amounting to 285 grains, or less than one-eighth of the whole quantity, instead of more than one-fifth, as in the Edinburgh Pharmacopœia.

Dr. Thomson has inadvertently omitted from the formula of the London Pharmacopœia the words "or as much as may be sufficient." He says,

"in our experiments, distilled vinegar of a specific gravity of 1·007 required 320 grains of the sesquicarbonate to saturate a pint." According to the table of the densities and strengths of acetic acid, quoted by Dr. Thomson, acid of sp. gr. 1·007 contains about 4·25 per cent. of real acid, a pint of which should saturate 433 grains instead of only 320 of the ammoniacal salt; suspecting, however, that this statement might have been copied from a former edition of the Dispensatory which had appeared before the introduction of the imperial pint, we referred to the eighth, and there we found it, and evidently meaning the wine pint.

*Ammoniæ Bicarbonas.*—This salt has been introduced into the Dublin Pharmacopœia, and Dr. Christison justly remarks that, it seems to be an unnecessary addition; he states, but on whose authority we know not, that it contains only one equivalent of water; Berzelius mentions that it contains two equivalents, and with him other chemists agree.

After mentioning this salt as containing ammonia, Dr. Christison adds, "or, according to the new theory of the constitution of the ammoniacal salts, it is an anhydrous compound of one equivalent of oxide of ammonium, and two of carbonic acid ($HN^4\ O + 2\ CO$);"—two mistakes occur in this formula, $HN^4$ should be $NH^4$, and 2 CO should be $2\ CO^2$—indeed, this latter mistake occurs four lines before; but, if we regard this salt as containing ammonia, it also contains two equivalents of water; and one of these must remain undecomposed on the theory of the formation of oxide of ammonium, and therefore its formula must be $HO,NH^4O + 2\ CO^2$. A still more recent view of the constitution of ammoniacal salts is that proposed by Dr. Kane, who considers ammonia as a compound of amidogen and hydrogen $NH^2,H$ or Ad H.

*Ammoniæ Spiritus.*—We admit the accuracy of Dr. Christison's statement that, in this preparation "the ammonia is obtained upon precisely the same chemical principles as for the watery solution." We cannot however agree with him, that the formula "requires no comment." In preparing the aqua ammoniæ, the College employ equal weights of hydrochlorate of ammonia and lime, the latter being nearly twice as much as absolutely requisite, but in this preparation the quantity of lime is even one-half greater than that of the ammoniacal salt—surely no reason can be assigned for this increased quantity.

*Antimonii Oxidum.*—The following is the formula of the Edinburgh Pharmacopœia for this preparation.

Take of Sulphuret of Antimony in fine powder, four ounces;
Muriatic Acid (commercial) one pint;
Water, five pints.

Dissolve the sulphuret in the acid with the aid of a gentle heat; boil for half an hour; filter; pour the fluid into the water; collect the precipitate on a calico filter; wash it well with cold water, then with a weak solution of carbonate of soda, and again with cold water till the water ceases to affect reddened litmus paper. Dry the powder over the vapour bath.

For reasons which do not appear, Dr. Christison has substituted *liquid* for *fluid*, *cloth* for *calico*, and *washings cease*, for *water ceases;* there seems to be no harm in the alteration, but the amendment is not equally obvious.

It is stated by Dr. Christison that "the quantity of muriatic acid used in the process appears large, being at least three times as great as is required to furnish the due proportion of chlorine for forming the sesquichloride. It is nevertheless necessary; Dr. Clark of Aberdeen informs me that he has in vain attempted to reduce the quantity."

We admit the accuracy of this statement as to the excess of muriatic acid, and although Dr. Clark is an authority from whom we hesitate to differ, yet direct experiment has assured us that his opinion is erroneous. We found that five fluidounces, or a quarter of a pint of muriatic acid of density 1160 dissolved two ounces of sulphuret of antimony, which, with an acid four per cent. weaker, is exactly double the Pharmacopœia quantity. The precipitated oxichloride was perfectly white, and we have no doubt but the acid would have dissolved more sulphuret; we are therefore quite satisfied that considerably more than half of the muriatic acid ordered by the Edinburgh College is entirely wasted.

The name given in the Dublin Pharmacopœia to the precipitate obtained by adding water to sesquichloride of antimony is Antimonii Oxydum nitromuriaticum, which Dr. Christison explains to be "sesquioxide of antimony, with some adhering chloride of antimony;" this however is neither a translation nor a correct description, for it is a definite crystallizable compound, or oxichloride of antimony, consisting, according to Professor Johnstone, of 9 equivalents of sesquioxide and 2 equivalents of sesquichloride of antimony; in p. 134, Dr. Christison again misstates its composition, calling it "dichloride of antimony." He further observes that "the process is productive according to the quality of the sulphuret used. Dr. Barker got $16\frac{1}{2}$ grains of the Dublin oxide from 20 grains of sulphuret; but I have got as much as 1534 from 1750." This is in the proportion of $17\frac{1}{2}$ from 20, being one grain more than obtained by Dr. Barker.

*Antimonium Tartarizatum.*—With respect to this preparation Dr. Christison remarks that "its name has been much tortured by reformers of pharmaceutic nomenclature. The original convenient term Tartar-emetic was successively changed to Tartarized antimony, and the Tartrate of antimony and potash; but now the London College has invented a newer designation still, which, according to the principles of nomenclature in its new Pharmacopœia, if it has meaning at all, implies the belief of the College that this double salt is a compound, not of tartaric acid with two bases, but of one base, oxide of antimony, with a compound acid, potassio-tartaric acid, of which potassium is one of the radicles. I am not aware that any chemist in recent times believes in the existence of such an acid."

Nor are we aware that any chemist ever thought of its existence till Dr. Christison "tortured" it into being; to show, however, that no such meaning has been attached to salts whose nomenclature is constructed on the same principle, we may refer to Dr. Thomson's Inorganic Chemistry, vol. 2, p. 792, where he describes a series of salts as *potash-tartrates*, and among them is *potash-tartrate of antimony*—the salt in question; in Brande's Manual we find (p. 917) *Potassio-sulphate of Chromium;* does Dr. Christison suppose that this last salt is imagined to contain *potassio-*

*sulphuric acid?* He may perhaps contend that Dr. Thomson uses the word *potash* and not *potassio*, and if he thinks that such plea will strengthen his criticism, we readily grant him all the support that he can derive from it.

We have already noticed Dr. Christison's statement, that the Antimonii Oxidum nitromuriaticum of the Dublin Pharmacopœia, is "sesquioxide of antimony with some adhering chloride of antimony;" and, in p. 146, the assertion is repeated that "in the process of the Edinburgh and Dublin Colleges, there is first obtained a sesquioxide of antimony with a little adhering chloride." In proof of the inaccuracy of this statement, we again refer to Professor Johnstone's Memoir, by which it appears "that the composition of the powder of algaroth, whether in the form of a dull grey sandy powder, or of regular crystals, is very constant," consisting, as he shows, of 74·5 per cent. of sesquioxide with 25·5 per cent. of sesquichloride. Now one-fourth can hardly be reckoned "a little."

The following are the directions of the Edinburgh Pharmacopœia for preparing Antimonii Potassio-tartras:

"Take of Sulphuret of Antimony, in fine powder, four ounces;
Muriatic acid (commercial) one pint;
Water, five pints;
Dissolve the sulphuret in the acid with the aid of a gentle heat; boil for half an hour; filter; pour the liquid into the water; collect the precipitate on a calico filter, wash it with cold water till the water ceases to redden litmus paper; dry the precipitate over the vapour bath.

Take of this precipitate three ounces;
Bitartrate of potash, four ounces and two drachms;
Water, twenty-seven fluidounces;
Mix the powders, add the water, boil for an hour, filter, and set the liquid aside to crystallize. The mother-liquor when concentrated yields more crystals, but not so free of [from] colour, and therefore requiring a second crystallization."

We shall first theoretically examine into the propriety of the assigned quantities of the bitartrate and oxichloride, which are four ounces or 1920 grains of the former, and three ounces or 1440 grains of the latter. Now by referring to the composition of crystallised tartarized antimony it will be seen that 189, or 1 equivalent of bitartrate is combined with 154 or two equivalents of sesquioxide, and consequently 1920 will require 1564; according to the analysis of oxichloride, already quoted, 100 contain 76·8 of metal, equivalent to 90 of sesquioxide of antimony; 1440 will therefore yield only 1296 of oxide instead of 1564 as required; the proportions of 100 of the salt to 80 of the oxichloride, which are the quantities of the Dublin College, are certainly preferable to 100 and 70, the proportions of the Edinburgh College; indeed, in our experiments made many years since, it appeared that 90 of the oxichloride to 100 of the bitartrate were the best proportions to be employed; a slight excess, which remains for future use, seems requisite to ensure the perfect saturation of the acidulous salt.

Referring to the formulæ of the three Colleges, Dr. Thomson remarks that, "by all these methods, a little modified, crystallized tartar-emetic may be prepared;" this means, we presume, that without some modification, it cannot, which opinion is certainly at variance with fact, for without any alteration they will all yield the salt in question. We do not mean to assert that they might not be improved, but this is very different from insinuating that they are totally inefficient.

"With regard to the formula of the London College, it is," says Dr. T. "a bad modification of the Edinburgh process." The following extraordinary reason is given for this opinion, "the intention of the addition of the hydrochloric acid is to prevent the formation of sulphuret of potassium, and the presence of free potassa, in the residue of the ignition; but a chloride of antimony is also formed, which in crystallizing mixes with the crystals of the tartar-emetic, and requires many recrystallizations for their complete separation."

Now we will for a moment admit, what we do not believe, that chloride of antimony is formed, for only four fluidounces of muriatic acid are used with 24 ounces each of sulphuret of antimony and nitre, and of the acid a great portion is certainly occupied in the mode described by Dr. Thomson. In the Dublin Pharmacopœia, 100 parts of muriatic acid are employed with 20 of sulphuret of antimony, and yet Dr. Thomson says it is "to be regretted that it was not adopted by the London College." Now, if the small quantity of muriatic acid employed by the London College occasions the crystallization of chloride of antimony, what effect is produced by the larger quantity used by the Dublin College? The fact, however, is, and Dr. Thomson must be well acquainted with it, that when chloride of antimony comes into contact with water, it is decomposed into muriatic acid and oxichloride of antimony, and it is consequently quite impossible that it should crystallize with the tartarized antimony. We will only further remark, that if the badness of the London formula depends upon the accuracy of the above statement, then it is faultless.

*Liquor Arsenicalis.*—We shall, in the first place, notice some inaccuracies which Dr. Christison has committed, with respect to this important preparation, by attempting to compress the differing directions of the three Pharmacopœias into too small a space: and, that we may do no injustice either to Dr. Christison or the subject under discussion, we shall give separately the directions of each Pharmacopœia; they are as follows:—

London Pharmacopœia. *Liquor Potassæ Arsenitis.*

℞ Acidi Arseniosi in frustula triti,
Potassæ Carbonatis, singulorum grana octaginta,
Tincturæ Lavandulæ Compositæ fluidrachmas quinque,
Aquæ destillatæ octarium;

Acidum Arseniosum et Potassæ Carbonatem cum Aquæ octario dimidio in vase vitreo coque, donec liquentur. Liquori frigefacto adjice Tincturam Lavandulæ Compositam. Denique adjice insuper Aquæ destillatæ quantum satis sit, ut mensuram octarii accuratè impleat.

Dublin Pharmacopœia. *Liquor Arsenicalis.*

℞ Arsenici Oxydi Albi Sublimati in pulverem triti,
Potassæ Carbonatis e Tartaro singulorum grana sexaginta,
Spiritûs Lavandulæ compositæ mensurâ drachmas quatuor,
Aquæ distillatæ *mensurâ* semilibram.

Arsenici Oxydum et Potassæ Carbonas simul in Aqua vase vitreo coque, donec solvatur Oxydum. Liquori frigefacto adjice Spiritum Lavandulæ compositum, et aquæ distillatæ quantum satis sit ut totius quantitas ex Librâ unâ *mensurâ* constet.

EDINBURGH PHARMACOPŒIA. *Liquor Arsenicalis.*

Take of White Arsenic in powder, and Carbonate of Potash, of each, four scruples;
Compound Tincture of Lavender, five fluidrachms;
Water, one pint;

Dissolve the oxide and carbonate together in half the water, with the aid of heat; filter, if necessary; add the tincture to the liquid when cold, and then dilute it with water till the whole measure one pint.

We may remark that there are some slight inaccuracies in these directions; first, the words *of each* should have been merely *each,* the word *of* having been employed before; secondly, no oxide is mentioned, and yet the directions are to dissolve the *oxide,* they should have been to dissolve the white arsenic; thirdly, in so important a preparation distilled water might have been ordered, without subjecting the College to the charge of incurring needless expense; fourthly, the Edinburgh Pharmacopœia contains a compound *Spirit* not *Tincture* of Lavender.

The directions of the three Colleges as above given, are thus compressed by Dr. Christison, in page 180 of his Dispensatory:

LIQUOR ARSENICALIS, *E. D.* LIQUOR POTASSAE ARSENITIS, *L.*

*Solution of arsenite of potass with excess of alkali.*

PROCESS, *Edin. Lond. Dub.* Take of

Powder of white arsenic, eighty grains (60, D.);

Carbonate of potash, 80 (60,D.) grains;

Compound tincture of lavender, (four, D,) five fluidrachms;

Distilled water, a pint (old wine m. D.)

Dissolve the white arsenic and carbonate of potash in half a pint of the water by boiling them in a glass vessel: Filter if necessary: Add the tincture when the solution has cooled, and then distilled water till the whole measures a pint.

The reader cannot fail to observe that the formula given by Dr. Christison does not contain all the peculiar directions of any one College, while it comprehends directions which do not belong to some of them: for example, the Edinburgh College does not direct distilled water to be used, which Dr. Christison's formula does; the London College directs, and for an obvious reason, not powdered arsenious acid, but small fragments; the Dublin College directs carbonate of potash from tartar, which, though Dr. Christison may think useless, the authors of that Pharmacopœia may not, and, in directions purporting to have originated with them, it ought to have been inserted. Compound Tincture of Lavender is not to be found by that name in the Dublin and Edinburgh Pharmacopœias, they term it compound Spirit of Lavender. There are other circumstances which must occur to any one who will make the comparison, proving that the formulas of the three Colleges should have been given at length; the only very material mistake is, however, in omitting at the end of the condensed formula (old wine m. D.) to show that the measure to which the Dublin preparation is to be made up, is the old wine and not the imperial pint.

*Solutio Barytæ Muriatis.*—With respect to this preparation Dr. Christison remarks that, "for no apparent reason, the London College has diluted the pre-existing solutions of the Colleges with nearly two volumes of water;" meaning of course the Dublin and Edinburgh Colleges, for the London Pharmacopœia, until the present edition, did not contain any formula for this solution.

On examination we certainly find that the former Dublin and Edinburgh Pharmacopœias contain directions for preparing the solution by dissolving one part of the salt in three parts of water; but it is a curious circumstance that Dr. Christison should have forgotten that what the London College has done "for no apparent reason," had been followed by the Edinburgh College; the London formula having been actually adopted in the present Edinburgh Pharmacopœia.

*Bismuthum Album.*—This is the name bestowed on this preparation by the Edinburgh College, because Dr. Christison says it "has resolved to avoid if possible the fluctuations of chemical language by adopting one of its old names, and a very convenient one, that of White Bismuth;" why then have not the College also adopted the name of white lead, certainly as old and as convenient a name as carbonate of lead?

With respect to tests the Edinburgh College have considered it sufficient to state that white bismuth "forms a colourless solution with nitric acid, and without effervescence;" the following substances, therefore, resemble it—hydrate of lime, magnesia, hydrate of lead and oxide of zinc.

The following observations, seemingly intended to have been appended to the remarks on Bismuth, are made by Dr. Christison in concluding those on Bismuthum Album: "the only officinal preparation is the *Pulvis bismuthi albi,* meaning, we presume, *Bismuthum album; P. bismuthi disnitratis* L. meaning *Bismuthi trisnitras;* and *P. bismuthi subnitratis* D. meaning *Bismuthi subnitras.*

*Creta Preparata.*—The method of testing the purity of this substance by the Edinburgh College, is the following: "A solution of 25 grains in ten fluidrachms of pyroligneous acid, when neutralized by carbonate of soda, and precipitated by 32 grains of oxalate of ammonia, continues precipitable after filtration by more of the test."

In the Materia Medica it is stated that 100 minims of pyroligneous acid "neutralize at least 53 grains of carbonate of soda," therefore 10 fluidrachms or 600 minims, must neutralize 318 of the carbonate, which are equivalent to 110 of carbonate of lime, consequently 4.4 times the requisite quantity of acetic acid is ordered for dissolving the carbonate of lime, and hence the necessity of employing nearly ten times its weight of carbonate of soda to saturate the excess of acetic acid, before the oxalate of ammonia is added; Dr. Christison says, that the excess of acetic acid is useful, but in what way we are utterly at a loss to conjecture.

It is further to be observed that the equivalent of oxalate of ammonia is 62, one half of which will of course decompose 25, or half an equivalent of carbonate of lime, whereas the College state that 32 are insufficient for this purpose.

Dr. Christison goes indeed still farther, and states that 32 of Oxalate of Ammonia will leave a little lime unprecipitated if there be 90 per cent. of pure carbonate of lime in the chalk, meaning that 32 of oxalate of ammonia are incapable of decomposing a solution of 22.5 of chalk, whereas they are capable of decomposing very nearly 26.

*Calcis Murias.*—The Edinburgh College has ordered this salt to be crystallized, which is a very inconvenient form, for in hot weather it fuses in its water of crystallization: it is, moreover, incorrectly stated that "a solution of 76 grains in one fluidounce of distilled water, precipitated by 49 grains of oxalate of ammonia, remains perceptible by more of the test." Dr. Christison states its composition to be chlorine 35.42, calcium 20.1, and water 54, making its equivalent 109.52, and requiring one equivalent, or 62 of oxalate of ammonia for decomposition, consequently 76 grains will require scarcely 43 grains of oxalate of ammonia, instead of more than 49, as stated by the Edinburgh College; so that this latter quantity is sufficient for more than 86 of the crystallized chloride, instead of less than 76 as asserted.

*Ferri Oxidum Nigrum.*—This is thus directed to be prepared in the Edinburgh Pharmacopœia:

Take of Sulphate of Iron, six ounces;
Sulphuric Acid, (commercial) two fluidrachms and two fluidscruples;
Pure Nitric Acid, four fluidrachms and a-half;
Stronger Aqua Ammoniæ, four fluidounces and a-half;
Boiling water, three pints.

Dissolve half the sulphate in half the boiling water and add the sulphuric acid; boil; add the nitric acid by degrees, boiling the liquid after each addition briskly for a few minutes. Dissolve the rest of the sulphate in the rest of the boiling water; mix thoroughly the two solutions; and immediately add the ammonia in a full stream, stirring the mixture at the same time briskly. Collect the black powder on a calico-filter; wash it with water till the water is scarcely precipitated by solution of nitrate of baryta; and dry it at a temperature not exceeding 180°.

It may be first observed, that a *fluidscruple*, though here directed to be employed, is not admitted by the College in their "system of measures," p. xv.; and this, if we mistake not, is the only occasion on which it is adopted.

The Edinburgh College have given three translations of Aqua Ammoniæ fortior, namely, *Concentrated aqueous solution of ammonia, Strong ammonia,* and *Stronger solution of ammonia;* but so much do they appear to approve of what they term a "patchwork" nomenclature, that neither English nor Latin appears to have pleased them on this occasion, and perhaps on some others, and therefore, in defiance of all propriety, a name is used composed of both languages—*Stronger Aqua Ammoniæ.* We are quite at a loss to imagine what advantage is gained by using the stronger solution of ammonia in this preparation; it is less economical in preparing than the weaker, and Dr. Christison admits that "it cannot be poured from one vessel to another, or kept unless during Winter, and in very well closed bottles, without parting with some of its ammoniacal gas; and hence the commercial *Aqua ammoniæ fortior* commonly ranges between 886 and 910." The latter part of this statement shows, therefore, that it is apt to lose about one-fourth of its ammonia.

It appears also, that whereas *commercial* sulphuric acid is sufficiently pure to dissolve sesquioxide of iron, *commercial* nitric acid is not sufficiently so to convert protoxide into sesquioxide, and accordingly we find *pure* nitric acid directed for that purpose; it is, however, but candid to admit that, on a similar occasion, in the preparation of *Ferrugo*, commercial nitric acid is directed to be employed.

The oxide obtained by this process is explained in the Pharmacopœia and by Dr. Christison to be the "ferroso-ferric oxide, (Berzelius,) a compound of protoxide and sesquioxide of iron," but it is not the ferroso-ferric oxide of Berzelius.

In his *Traité de Chimie*, Berzelius states the octohedral magnetic iron ore to be the ferroso-ferric oxide; this is composed of one equivalent of protoxide 36, and two equivalents of sesquioxide 80; this, therefore, contains one more equivalent of sesquioxide than the Edinburgh preparation, which consists of one equivalent of each. In his *Table Synoptique* Berzelius mentions another ferroso-ferric oxide, which is composed of one-and-a-half equivalent of protoxide 54, and one of sesquioxide 40, and it consequently contains half an equivalent more of protoxide than the ferri oxidum nigrum; it is therefore evident that this preparation is not either of the ferroso-ferric oxides of Berzelius. Dr. Christison afterwards, and more correctly, states that, according to the analysis of Wohler, this compound may be regarded as composed of an equivalent of protoxide 36, and one of sesquioxide 40; indeed, it is evident that this must be the case, for equal quantities of iron are precipitated together, one-half being protoxide and the other sesquioxide, and these oxides, to adopt Dr. Christison's rather singular description, "unite at once in the act of separation."

In copying this formula, Dr. Thomson has stated "*four fluidounces and a half*" of nitric acid, instead of "*four fluidrachms and a half.*" With respect to the composition of the ferri oxidum nigrum, he says, "this is an admixture of two oxides, namely, 2 eqs.=72 of the protoxide, and 1 eq.=40 of the sesquioxide, and 2 eqs.=18 of water; its formula being $1\frac{1}{2}$ Fe. O.+Fe.$^{1}$ O$^{1}$+2 HO. In this statement Dr. Thomson has reversed the equivalents of protoxide and sesquioxide; they being respectively 1 to 2, and not 2 to 1; moreover, when this correction is made, it does not represent the composition of the Ferri Oxidum Nigrum, as we have already shown; if we understand the formula given by Dr. T., it means a compound of one equivalent of an oxide of iron, consisting of $1\frac{1}{2}$ equiv. of iron, and 1 equiv. of oxygen, which does not exist, combined with 1 equiv. of protoxide of iron; so that an incorrect composition is illustrated by an incorrect formula.

[*To be concluded in our next Number.*]

