# Errata

**Published:** 1845-01

**Authors:** 


					errata.
page !'-ne ? /r?m top>f?r Perceptible, read precipitahle.

				

