# Erratum

**Published:** 1845-04

**Authors:** 


*Erratum.*—P. 218, line 8 from top, for *perceptible* read *precipitable.*

